# Predicting Verbal Learning and Memory Assessments of Older Adults Using Bayesian Hierarchical Models

**DOI:** 10.3389/fpsyg.2022.855379

**Published:** 2022-04-14

**Authors:** Endris Assen Ebrahim, Mehmet Ali Cengiz

**Affiliations:** ^1^Department of Statistics, Faculty of Science and Literature, Institute of Graduate Studies, Ondokuz Mayis University, Samsun, Turkey; ^2^Department of Statistics, College of Natural and Computational Sciences, Debre Tabor University, Gondar, Ethiopia

**Keywords:** predicting, Hamiltonian Monte Carlo, Verbal Learning Test, hierarchical, model

## Abstract

Verbal learning and memory summaries of older adults have usually been used to describe neuropsychiatric complaints. Bayesian hierarchical models are modern and appropriate approaches for predicting repeated measures data where information exchangeability is considered and a violation of the independence assumption in classical statistics. Such models are complex models for clustered data that account for distributions of hyper-parameters for fixed-term parameters in Bayesian computations. Repeated measures are inherently clustered and typically occur in clinical trials, education, cognitive psychology, and treatment follow-up. The Hopkins Verbal Learning Test (HVLT) is a general verbal knowledge and memory assessment administered repeatedly as part of a neurophysiological experiment to examine an individual’s performance outcomes at different time points. Multiple trial-based scores of verbal learning and memory tests were considered as an outcome measurement. In this article, we attempted to evaluate the predicting effect of individual characteristics in considering within and between-group variations by fitting various Bayesian hierarchical models *via* the hybrid Hamiltonian Monte Carlo (HMC) under the Bayesian Regression Models using ‘Stan’ (BRMS) package of R. Comparisons of the fitted models were done using leave-one-out information criteria (LOO-CV), Widely applicable information criterion (WAIC), and K-fold cross-validation methods. The full hierarchical model with varying intercepts and slopes had the best predictive performance for verbal learning tests [from the Advanced Cognitive Training for Independent and Vital Elderly (ACTIVE) study dataset] using the hybrid Hamiltonian-Markov Chain Monte Carlo approach.

## 1. Introduction

Verbal learning and memory tests are highly varied among older-aged adults due to various influences. Early cognitive intervention in older adults is a critical program to reduce the future risk of dementia (Thomas et al., 2019). The efficacy of the Chinese form Hopkins Verbal Learning Test (HVLT) for screening dementia and mild cognitive impairment in a Chinese population showed that HVLT scores were affected by age, education, and sex (Shi et al., 2012). The dataset of Advanced Cognitive Training for Independent and Vital Elderly (ACTIVE) study consists of two hierarchies in which four different repeated measures are nested within each participant (Luo and Wang, 2014). The outcome measures of the cognitive training interventions were the total HVLT from three learning trials and the baseline measure (Gross, 2011).

Bayesian logistic and hierarchical probit models of accuracy data that allow two levels of mixed-effects in repeated-measures designs have been implemented. The Bayes factor through the Bayesian information criterion estimate and the Widely applicable information criterion (WAIC) model selection techniques were used (Song et al., 2017). Duff (2016) used stepwise regression model to scrutinize the effect of age, education, and gender on HVLT scores in 290 cognitively intact older adults. The study revealed that age was negatively correlated with the HVLT score, while education status was positively correlated. Moreover, there were fewer gender differences among four repeatedly measured verbal learning tests (Lekeu et al., 2009).

Another study showed that besides capabilities through training, personal characteristics like age, unmarried status, and lower occupational cognitive requirements increased the likelihood of cognitive risk (Silva et al., 2012). Higher educational levels and active engagement in exercise may contribute to cognitive reserve and have a protective effect on cognitive decline in late life (Shen et al., 2021).

Gender effects on neuropsychological performance were negligible when the age and educational status of elderly people were controlled (Welsh-Bohmer et al., 2009). Recently, the Markov chain Monte Carlo (MCMC) methods have been widely used to generate samples from complicated and high-dimensional distributions (Hadfield, 2017). Among all Bayesian computational methods, the Hamiltonian Monte Carlo (HMC) (Almond, 2014) approach is the most efficient for approximating complex data structure models and converges faster than the traditional Metropolis-Hastings and Gibbs methods (Kruschke and Vanpaemel, 2015). The common MCMC approaches show poor performance and tremendously slow convergence in complex parameter structures (Yao and Stephan, 2021).

The HVLT is the ultimate in situations calling for multiple neuropsychological assessments (Benedict et al., 1998). Classical statistical inferences and single-level models have limitations for predicting naturally nest data. Bayesian hierarchical models (Congdon, 2020) were able to predict verbal learning test and memory scores from baseline personal characteristics, such as age, gender, cognitive status [mini-mental state exam (MMSE) score], years of education, and participants’ booster training and reasoning ability measured by training progress (Kuslansky et al., 2004).

In Bayesian inference, the WAIC, the leave one out information criterion (LOO-IC), and K-fold cross-validation (K-fold-CV) are recently developed measures of complexity penalized fitting models (Almond, 2014; Sivula et al., 2020). In this article, model comparisons and model selections were performed using these three methods under the Bayesian Regression Models using ‘Stan’ (BRMS) package of R (Bürkner, 2018). In most cases, WAIC and LOO-IC showed a slight preference for the random slope model over other models (Bürkner, 2018). However, the general model selection principle shows to choose the null model when diffuse priors are used in the parameters to be included or rejected by the algorithms (Liu, 2000). Therefore, in this article, we used the HMC approach to fit the three different Bayesian hierarchical models and select the best predictive model.

## 2. Materials and Methods

### 2.1 Data and Variables

The ACTIVE study was a randomized controlled trial conducted in 1999–2001 at six diverse research centers in the United States and organized by the New England Research Institutes (NERI). A total of 1,575 purposively selected older adults were included in this study (Willis et al., 2015), in which 26% of the participants were African American. The ACTIVE dataset accessed from the study of Willis et al. (2015) has 13 variables. However, this modeling paper used six explanatory variables, and the dependent variable HVLT is used as repeated measures of learning tests and memory ability. In this dataset, HVLT has four different repeated measurement scores doi: 10.3886/ICPSR04248.v3.

### 2.2 Bayesian Hierarchical Model for Repeated Measures Data

Suppose **X** is the matrix of explanatory variables, and **Y** is the outcome variable that is the Total Hopkins Verbal Learning Test Score (***THVLTS***). Besides the classical statistics, a more flexible Bayesian model is required that can accommodate the varying correlation between covariates and independent variables that occur in repeated measures-type longitudinal data. The general form of the Bayesian hierarchical model for repeated measures data can be expressed as:


(1) $${\text{Y}}_{N\times 1}=\underset{fixedeffects}{\underbrace{{\text{X}}_{N\times p}\beta_{p\times 1}}}+\underset{randomeffects}{\underbrace{{\text{Z}}_{N\times mq}{\text{U}}_{mq\times 1}}}+\underset{residuals\left(errorterm\right)}{\underbrace{\varepsilon_{N\times 1}}}$$

Where **Y** denotes the vector ${\left(y_{1}^{\prime },y_{2}^{\prime },\ldots y_{m}^{\prime }\right)}^{\prime }$ of outcome variable; β denotes a vector of fixed effects parameters; **U** denotes a vector ${\left(u_{1}^{\prime },u_{2}^{\prime },\ldots u_{m}^{\prime }\right)}^{\prime }$ of associated random effects (*specifictoeachsubject*); **X** is a matrix of covariates (explanatory variables); **Z** denotes a block diagonal matrix of covariates for the random effects as a complement of **X** embraced of m blocks that each block has *n*_*i*_ × *q* dimension matrix and ε denote a column vector of residuals. We assumed that the random effects **U**∼N(0_*d*_, **Ω**) and the residuals $\varepsilon ∼N\left(0_{n_{i}},\text{R}=\sigma_{e}^{2}\right)$. Where **U** and ε are independently distributed. Based on the unknown vector of φ_**Ω**_ and φ_**R**_, the unknown random effects in **Ω** and **R** can be written as **Σ** = (φΩ, φ_R_) (Laird and Ware, 1982).


$$Y_{i}=X_{i}^{\left(F\right)}\beta^{\left(F\right)}+X_{i}^{\left(R\right)}\beta^{\left(R\right)}+\varepsilon_{i}$$

$$\left(\begin{array}{c} Y_{i1}\\ Y_{i2}\\ \vdots \\ Y_{in_{i}} \end{array}\right)=\left(\begin{array}{cc} 1t_{i1} & \\ 1t_{i2} & \\ \vdots \;\vdots & \\ 1t_{in_{i}} & \end{array}\right)\left(\begin{array}{c} a_{11}\\ a_{21} \end{array}\right)+\left(\begin{array}{cc} 1t_{i1} & \\ 1t_{i2} & \\ \vdots \;\vdots & \\ 1t_{in_{i}} & \end{array}\right)\left(\begin{array}{c} u_{1i}\\ u_{2i} \end{array}\right)+\left(\begin{array}{c} \varepsilon_{i1}\\ \varepsilon_{i2}\\ \varepsilon_{i3}\\ \vdots \\ \varepsilon_{in_{i}} \end{array}\right)$$

Where **X** is divided into two columns corresponding to fixed effects and a corresponding random effects design matrix denoted as $X_{i}^{\left(F\right)}$ and $X_{i}^{\left(R\right)}$, respectively. And the parameters are divided into fixed effects β^(*F*)^ and random effects β^(*R*)^ = **U**. *Cov*(*u*_*i*_,*u*_*i*_) = *Var*(*u*_*i*_) = **Ω** and


$$\left[\begin{array}{c} u\\ \text{β} \end{array}\right]∼MVN\left(\left(\begin{array}{c} {\text{μ}}_{u}\\ {\text{μ}}_{\text{β}} \end{array}\right),\left(\begin{array}{cc} {\text{σ}}_{u}^{2} & {\text{ρσ}}_{u}{\text{σ}}_{\text{β}}\\ {\text{ρσ}}_{u}{\text{σ}}_{\text{β}} & {\text{σ}}_{\text{β}}^{2} \end{array}\right)\right)$$

It can be assumed that the hyperparameters of both the intercept and the coefficient/slope model have uniform hyper-prior distributions with appropriate assumptions for the parameters μ_*u*_, μ_β_, σ_*u*_, σ_β_ *ve* ρ. Then, the mathematical form of the three possible Bayesian hierarchical models (Nalborczyk and Vilain, 2019) for predicting the verbal learning and memory test with two (group/subject and time) random effects (Hilbe, 2009) can be written as follows:

#### Model 1: Null Model

Here, the model is fitted by varying the intercept without including any predictor variable. Thus, this model shows the overall within and between-subject variations of the outcome variable (Goldstein et al., 2009).


$$THVLTS_{i}∼Normal\left(\mu_{i},\sigma_{e}\right),i=1,2,3,\ldots n$$

$$\mu_{i}=\alpha +\alpha_{subject\left[i\right]}+\alpha_{time\left[i\right]}$$

$$\alpha_{subject}∼Normal\left(0,\sigma_{subject}\right)$$

$$\alpha_{time}∼Normal\left(0,\sigma_{time}\right)$$

α∼Normal(0,10)

$$\sigma_{subject}∼HalfCauchy\left(0,1\right)$$

$$\sigma_{time}∼HalfCauchy\left(0,1\right)$$

$$\sigma_{e}∼HalfCauchy\left(0,1\right)$$

#### Model 2: Varying Intercept Model

Here, the BRMS command is fitted in R with varying intercepts for both clusters (i.e., participating subjects) and repeated measures (i.e., measurement time point) by including all predictor variables in the model. Thus, this model can be called a random intercept and fixed slope model (McGlothlin and Viele, 2018).


$$THVLTS_{i}∼Normal\left(\mu_{i},\sigma_{e}\right)$$

$$\mu_{i}=\alpha +\alpha_{subject\left[i\right]}+\alpha_{time\left[i\right]}+\beta X_{i}$$

$$\alpha_{subject}∼Normal\left(0,\sigma_{group}\right)$$

$$\alpha_{time}∼Normal\left(0,\sigma_{time}\right)$$

β∼Normal(0,10)

α∼Normal(0,10)

$$\sigma_{subject}∼HalfCauchy\left(0,1\right)$$

$$\sigma_{time}∼HalfCauchy\left(0,1\right)$$

$$\sigma_{e}∼HalfCauchy\left(0,1\right)$$

#### Model 3: Varying Slopes

Here, we can focus on examining the dependence between the random intercepts and the random coefficients (Bafumi and Gelman, 2011). In this case, we are interested in whether the effects of *age* and *reasoning skill* have correlations with variations in verbal and memory test skills measured by trail scores.


$$\text{T}HVLTS_{i}∼Normal\left(\mu_{i},\sigma_{e}\right)$$

$$\mu_{i}=\alpha +\alpha_{subject\left[i\right]}+\alpha_{time\left[i\right]}+\beta X_{i}+\left(\beta +\beta_{subject}\right)X_{i})$$

$$\left[\begin{array}{c} \alpha_{subject}\\ \beta_{subject} \end{array}\right]∼MVN(\left[\begin{array}{c} \alpha \\ \beta \end{array},S\right]$$

$$S=\left[\left(\begin{array}{cc} {\text{σ}}_{\text{α}} & 0\\ 0 & {\text{σ}}_{\text{β}} \end{array}\right)R\left(\begin{array}{cc} {\text{σ}}_{\text{α}} & 0\\ 0 & {\text{σ}}_{\text{β}} \end{array}\right)\right]=\left[\begin{array}{cc} {\text{σ}}_{\text{α},subject}^{2} & {\text{σ}}_{\text{α}}{\text{σ}}_{\text{β}}\text{ρ}\\ {\text{σ}}_{\text{α}}{\text{σ}}_{\text{β}}\text{ρ} & {\text{σ}}_{\text{β},subject}^{2} \end{array}\right]$$

$$\alpha_{subject}∼Normal\left(0,\sigma_{subject}\right)$$

$$\alpha_{time}∼Normal\left(0,\sigma_{time}\right)$$

β∼Normal(0,10)

α∼Normal(0,10)

$$\sigma_{\alpha ,subject}∼HalfCauchy\left(0,1\right)$$

$$\sigma_{time}∼HalfCauchy\left(0,1\right)$$

$$\sigma_{e}∼HalfCauchy\left(0,1\right)$$

$$\sigma_{\beta subject}∼HalfCauchy\left(0,1\right)$$

$$\text{R}∼LKJ_{corr}\left(2\right)$$

Where **S** is the covariance matrix, $R=\left(\begin{array}{cc} 1 & \text{ρ}\\ \text{ρ} & 1 \end{array}\right)$ is the corresponding correlation matrix, and ρ is the association between intercepts and coefficients used in the calculation of **S**. The prior matrix **R** is the LKJ-correlation (Lewandowski et al., 2009) with a parameter ζ(*zeta*) which regulates the strength of the association.

**FIGURE 1:**
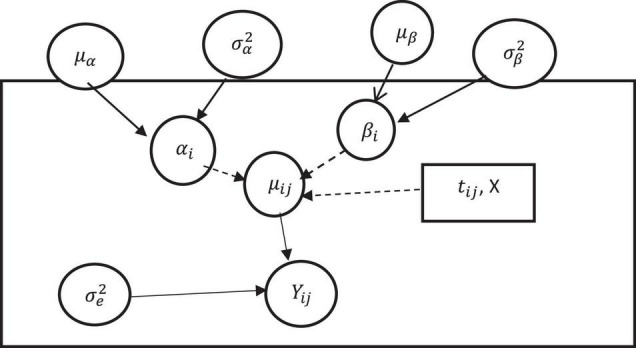
A varying intercept and slope model (Bayesian Framework).

As shown in Figure 1 above, each component of the mixed effect model appears in the graph as a node. The dotted arrows represent deterministic (fixed) dependencies between the parameters (e.g., from β to μ_*ij*_), whereas the solid arrows represent probabilistic (random) dependencies (e.g., from $\sigma_{e}^{2}$ to *Y*_*ij*_) (Bürkner, 2018). The hyper-parameters of the varying both intercept and slope model (μ_α_, μ_β_, σ_α_, σ_β_, and ρ) can be assumed to have hyper-prior distributions with appropriate assumptions for the parameters (Liu, 2016; Congdon, 2020).

### 2.3 Bayesian Information Criterion for Model Comparison and Selection

#### Watanabe’s Widely Applicable (WAIC)

WAIC (Watanabe, 2010) could be achieved as an improvement over the divergence-based information criterion (DIC) for Bayesian models. The deviation term used in the calculation of the WAIC is Log-Point Based -Requires Predictive-Density (LNTTY). LNTTY is calculated as:


$$\text{LNTTY}=\sum_{i=1}^{N}\log\int p\left(y_{i}|\theta \right)\times p_{post}\left(\theta \right)d\theta$$

The whole *p*_*post*_(θ) is the posterior distribution used in the calculation of LNTTY. Similar to LNTTY, WAIC’s penalty term is purely Bayesian and is computed as:


$$p_{WAIC}=\sum_{i=1}^{N}Var_{post}\left(\log p\left(y_{i}|\theta \right)\right)$$

Where *p*_*WAIC*_ is the penalty term which is the variance of the log-predictive-density terms aggregated over N data points. Thus, the WAIC can be calculated as:


$$WAIC=-2LPPD+2p_{WAIC}$$

#### Leave-One-Out Information Criteria (LOO-CV)

Bayesian leave-one-out cross-validation (LOO-CV) is different from the WAIC. Because there is no penalty term in its calculation. LOO-CV can be computed as:


$$LOOIC=-2LPPD_{loo}=-2*\sum_{i=1}^{N}\log$$

$$\int p\left(y_{i}|\theta \right)\times p_{post\left(-i\right)}\left(\theta \right)d\theta$$

Where *p*_*post*(−*i*)_(θ) is the posterior distribution based on a sub-set of the data at point *i* from the dataset. LNTTY used *i^th^* data points to calculate both the posterior distribution and the parameter estimation. Here, in contrast, the log-pointwise predictive density (*LPPD*_*loo*_) is used the same for prediction only. Therefore, there is no need for a penalty term to correct potential bias by using the data twice (Vehtari et al., 2017).

#### K-Fold Cross-Validation

Sometimes, multiple Pareto Corrected Significance Sampling (PSIS-LOO) fails, and it takes too long to remodel in the iteration. Therefore, we can estimate LOO-CV using K-fold-CV by separating the data into completely random multiples, which leads to looking at each cross-validation estimate distinctly (Vehtari et al., 2018).

The Bayesian K-fold-CV partitions the dataset into k subsets *y*_*k*_(*k* = 1, 2, …, *K*). The Bayesian hierarchical model (BHM) generates each training dataset *y*_*k_e_*_ separately, which returns a *p*_*post*(*e*)_(θ) = *p*(θ|*y*_(*k*_*e*_)_) posterior distribution (Vehtari and Gelman, 2014). To preserve reliability with WAIC and LOO-IC, defining the predictive accuracy of every point in the dataset is essential. Therefore, the log-predictive distribution function is


$$\log p_{post\left(k.e\right)}\left(y_{ı}\right)-\log\int p_{pred}\left(y_{ı}|\theta \right)p_{post\left(k.e\right)}\left(\theta \right)d\theta ,i\epsilon k.$$

Using “**S**” simulations corresponding to a subset of k (usually K = 10) containing the *i^th^* data point and the posterior distribution *P*(θ|*y*_(*k*_*e*_)_). The overall estimate of the expected log point predictive density for a new dataset is determined as follows:


$$\hat{elpd_{val}}=\sum_{i=1}^{n}{\hat{lpd}}_{i}=\sum_{i=1}^{n}\log\left(\frac{1}{S}\sum_{s=1}^{S}p\left(y_{i}|\theta^{k,s}\right)\right)$$

Therefore, a point estimate of the k-fold value is the sum of the iterative folds from the data points.

### 2.4 The Hamiltonian Monte Carlo Algorithm in Bayesian Regression Models Using ‘Stan’ Package of R

Similar to Gibbs sampling, HMC practices a proposal distribution that changes subject to the recent location in the parameter space (Liu, 2000). However, unlike the Gibbs algorithm, HMC does not rely on computing the conditional posterior distribution of parameters and sampling from it (Mai and Zhang, 2018). HMC has two advantages over other MCMC methods: little or no autocorrelation of the samples and fast mixing, i.e., the chain converges to the distribution immediately (Nalborczyk and Vilain, 2019). Therefore, it is the best approach for continuous distributions with low (auto) correlation and low rejection of samples.

When the model parameters are continuous rather than discrete, HMC, also known as Hybrid Monte Carlo, can overpower such random walk behavior using a clever scheme of supplementary variables that converts the tricky of sampling from the targeted function into the simulating Hamiltonian dynamics (Britten et al., 2021). HMC is an MCMC algorithm that avoids the random walk behavior and sensitivity to correlated parameters that outbreak other MCMC approaches by performing a series of steps informed by first-order gradient information (Hilbe, 2009).

The HMC algorithm is based on the Hamiltonian (total energy) calculating the trajectory for a time *t* = 0, …, *T* and then taking the final position *X*(*T*) = *X*_*n*+1_.

The steps of the algorithm are as follows:

HMC algorithm

1. Choose a starting point and a velocity distribution θ_0_ = *X*_0_*q*(*v*)
2. for *n* = 0, …
3. Set the initial position as *X*(*t* = 0) = *X*_*n*_
4. Draw a random initial velocity, *v*(*t* = 0)∼*q*(*v*);
5. Integrate the orbit numerically with the total energy for some time (use the Leapfrog method):

H(X,v)=U(X)+K

=-log⁡p(X)-log⁡q(v)T

6. Calculate the probability of acceptance:

$$\alpha \left({\text{X}}_{n+1},{\text{X}}_{n}\right)=min\left\{1,\frac{exp\left[-H\left({\text{X}}_{n+1},{\text{v}}_{n+1}\right)\right]}{exp\left[-H\left({\text{X}}_{n},{\text{v}}_{n}\right)\right]}\right\}$$

7. Set *X*_*n*+1_ = *X*(*t* = *T*)
8. Increment

## 3. Results

In practice, the three basic Bayesian hierarchical models have been fitted in BRMS default settings, and population-level (fixed) effects and subject-level (random) effects were obtained (Luo et al., 2021). All three models (Models 1, 2, and 3) had both fixed and random (mixed) parts but with different estimated parameter types. In the result, the estimate shows the posterior mean and Est. Error is the *SD* for each parameter. Model convergence was achieved well enough both the bulk effective sample size (Bulk_ESS) and the tail effective sample size (Tail_ESS) for the 95% CIs were adequate (Vehtari et al., 2017). In general, every parameter is summarized using the posterior distribution’s mean (“Estimate”) and *SD* (“Est. Error”), as well as two-sided 95% credible intervals as lower and upper bounds based on quintiles.

Table 1 of the fixed effects shows that the posterior mean verbal testing score was estimated to be 26.33 with an *SD* of 0.73. The 95% credible interval shows that the posterior distribution mean (intercept) was significant. On the other hand, the random effect showed significant verbal score test variation between groups (participant subjects) and within-subjects (between different measurements of different time points). Thus, according to the null model, the HVLT score showed more between-group/subject variation than within-group (between repeated measurements) variation.

**TABLE 1:** Results from the fitted null model: Model 1.

Outcome variable	Covariates	Estimate	Est. Error	Bulk_ESS	Tail_ESS	$\hat{\text{R}}$	95% CI	
	Fixed effects						Lower	Upper
Total hopkins verbal learning test score (THVLTS)	Intercept	26.3312	0.7331	1371	1875	1.01	24.8501	27.7214
	Random Effects						Lower	Upper
	σ_*intercept*,*subject*_	4.3105	0.0852	810	1450	1.00	4.1524	4.4751
	σ_*intercept*,*time*_	1.3035	0.6456	2047	2429	1.00	0.5754	3.0562
	σ_*e*_(*sigma*)	3.1134	0.0256	3315	3296	1.01	3.0462	3.1662

Table 2 showed that the coefficient of booster training was positive with a zero overlapping 95% CI. This indicates that, on average, there is little evidence that taking booster training increases elderly adults’ verbal learning and memory test scores by 0.1865, but the evidence-based on the data and random intercept model. On the other hand, adults’ years of education (edu) estimate was negative with a zero overlapping 95% CI. This negative estimate indicates that, on average, in the random intercept model, there is little evidence that increasing the years of education decreases elderly adults’ verbal learning and memory test scores by 0.0034 units.

**TABLE 2:** Results from the fitted varying intercept model: Model 2.

Outcome variable	Covariates	Estimate	Est. Error	Bulk_ESS	Tail_ESS	$\hat{\text{R}}$	95% CI	
	Fixed effects						Lower	Upper
Total hopkins verbal learning test score (THVLTS)	Intercept	9.2314	1.9411	1260	189	1.00	5.4712	12.9510
	Age	−0.1211	0.0212	926	1702	1.01	−0.1611	−0.0854
	Edu	−0.0034	0.0011	4139	2838	1.01	−0.0101	0.0042
	Booster	0.1865	0.1754	645	1838	1.00	−0.1511	0.5432
	Gender	2.6564	0.2015	910	1607	1.00	2.2752	3.0654
	Reason	0.1464	0.0112	980	1673	1.00	0.1310	0.4232
	MMSE	0.6012	0.0462	1032	2128	1.00	0.5013	0.7012
	Random effects						Lower	Upper
	σ_*intercept*,*subject*_	3.0312	0.0654	1146	2271	1.00	2.8845	3.1645
	σ_*intercept*,*time*_	1.2654	0.6572	1852	2121	1.00	0.5832	3.0812
	σ_*e*_(*sigma*)	3.1102	0.0312	4264	3029	1.00	3.0462	3.1761

**FIGURE 2:**
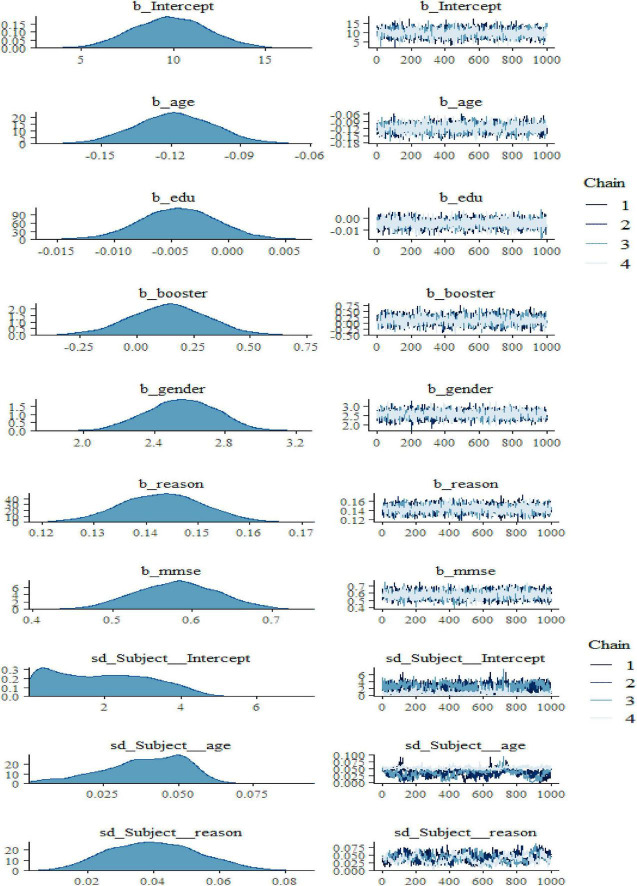
Bayesian hierarchical varying slope convergence diagnosis.

According to the predictive effects of each explanatory variable shown in Figure 2 and Table 3, taking booster training, age, and gender were the most influential factors affecting participants’ cognitive verbal test and memory ability. Table 3 reveals that there is also an adverse association between the intercepts and coefficients for reasoning ability, which implies reasoning ability has a large average score value showing additional variability by poor reasoning ability than by good reasoning ability. Nevertheless, it can be seen that the slope estimate of such a model is even further unreliable than that of the preceding models, as it can be clearly understood from the associated standard error and the size of the 95% CIs. Table 3 also showed that booster training had a significant positive predictive effect on elderly adults’ verbal learning and memory test scores. In contracts, adults’ years of education had a significant negative impact on elderly adults’ verbal learning and memory test scores.

**TABLE 3:** Results from the fitted varying slope mode: Model 3.

Outcome variable	Covariates	Estimate	Est. Error	Bulk_ESS	Tail_ESS	$\hat{\text{R}}$	95% CI	
	Fixed effects						Lower	Upper
Total hopkins verbal learning test score (THVLTS)	Intercept	9.8412	2.0602	3157	2918	1.00	5.8523	13.9344
	Age	−0.1211	0.0213	2846	2720	1.01	−0.1513	−0.0823
	Edu (education)	−0.0033	0.0012	5523	2770	1.00	−0.0122	0.0012
	Booster	0.1412	0.1703	3362	2876	1.00	−0.2145	0.4831
	Gender	2.5505	0.2004	3236	2866	1.01	2.1712	2.9331
	Reason	0.1444	0.0113	3087	2867	1.00	0.1313	0.4402
	MMSE	0.5803	0.0512	3256	3042	1.00	0.4822	0.6840
	Random effects						Lower	Upper
	σ_*intercept*,*subject*_	1.9222	1.2833	111	488	1.00	0.0724	4.3111
	σ_*intercept*,*time*_	1.3022	0.8004	2027	2270	1.00	0.5702	3.1343
	σ_*age*_	0.0424	0.0133	100	833	1.00	0.0123	0.0625
	σ_*reason*_	0.0405	0.0132	138	391	1.00	0.0212	0.0732
	*cor*_*Intercept*,*age*_	0.1033	0.4333	111	255	1.00	−0.7042	0.8303
	*cor*_*Intercept*,*reason*_	−0.3902	0.4204	100	388	1.00	−0.9011	0.6212
	*cor*_*age*,*reason*_	−0.5922	0.2645	519	1053	1.00	−0.9042	0.1407
	σ_*e*_(*sigma*)	3.1102	0.0333	3767	2748	1.00	3.0533	3.1710

**FIGURE 3:**
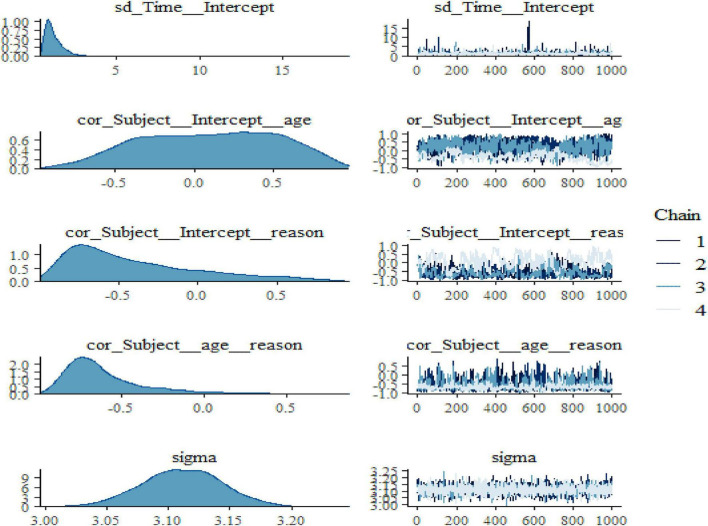
Bayesian hierarchical varying slope convergence diagnosis (Continuous).

We also noticed in Figure 2 and Figure 3 below that adding any term to the early model showed predictive performance improvements on the fitted models are ordered from Models 1 to 3 (full model). However, such a result may not be interpreted as a universal rule, subsequent adding extra terms to a unique model may also result in overfitting, which corresponds to a condition in which the fitted model is over-specified about the data, making the model good at clarifying the sample dataset but poor at predicting no observed data. The model convergence diagnosis plots are hairy caterpillars which showed the model converged. On the other hand, the models have well converged based on the estimated statistical values. This means that the R-hat $\left(\hat{R}\right)$ statistics were close to 1 and the (bulk and tail) ESSs values were sufficiently high when ESS > 100 was chosen as the cutoff (Vehtari et al., 2021). The majority of parameters still showed sufficiently high ESS values when more conservative cutoffs were chosen (i.e., ESS > 400 or even 1,000, see Zitzmann and Hecht, 2019).

Based on the fitted varying slope model, which accounted for six predictors from the data, fixed effects showed that age, gender, reasoning ability, and booster training were significant predictors of verbal learning and memory test scores, whereas random-effect showed that much of the variation in test scores occurred within-subjects (between measurement time points) than between subjects.

After we have built the three different models, it is necessary to identify relatively the best model that can be used to predict the outcome variable and make inferences. However, choosing the model that has the best predictive and a better fit on the actual data is complicated with diverse information criteria since all selected models on the actual data might not essentially achieve as fit on a different dataset. In its place, it is necessary to decide on a model that fits best in terms of predicting new data which had not been practiced.

In case of the non-existence of extra information, cross-validation methods such as WAIC and LOO-CV can be used. According to Table 4, the varying slope model has the lowest WAIC, LOO-IC, and 10-fold estimates. However, the difference is relatively small when we compare the difference in estimates of criteria for each model and the corresponding standard errors (in the column SE).

**TABLE 4:** Model comparisons based on predictive performance.

Model type	Model selection criteria from BRMS package					
	WAIC		LOO-IC		10-fold	
	Estimate	SE	Estimate	SE	Estimate	SE
Null model (Model 1)	33638.0	134.6	33744.1	136.2	33923.8	136.4
Varying Intercept model (Model 2)	33494.5	139.5	33574.9	140.6	33717.0	141.4
Varying slopes model (Model 3)	33488.4	141.8	33567.5	143.0	33685.2	140.8

Among the fitted models above, it looks like the final model (Model 3) in the HMC algorithm is the best model. Therefore, as a function of the six explanatory variables and the random coefficient for age and reasoning ability, Model 3 has the best predictive performance for the cognitive HVLT.

**FIGURE 4:**
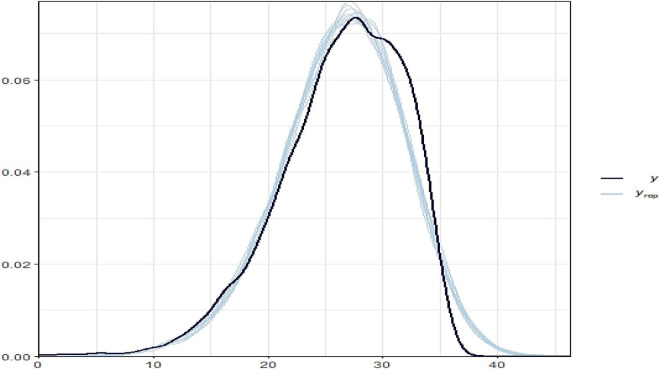
Bayesian hierarchical varying slope fitted model on the observed and predicted outcomes.

According to Figure 4, the varying slope and intercept model fit well and produced nearly identical posterior observed density and posterior predictive distribution plots of the outcome variable of THVLTS from the ACTIVE study.

**FIGURE 5:**
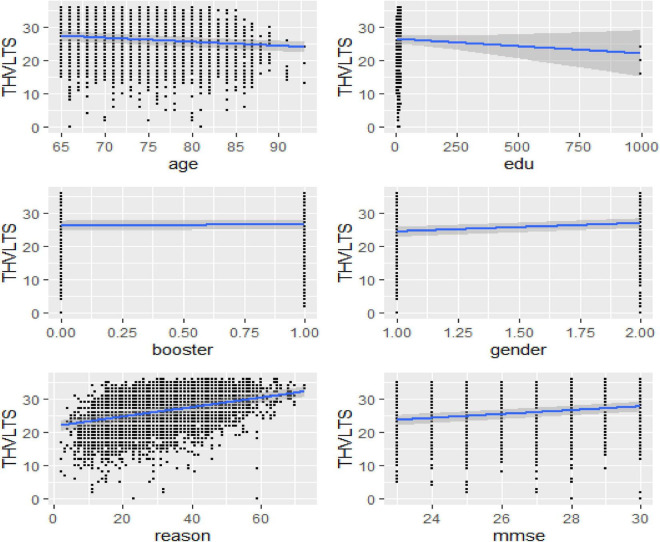
Bayesian hierarchical varying slope model marginal prediction effects.

Furthermore, the marginal effect of each predictor variable revealed (Figure 5) that age and reasoning skills are the most significant explanatory variables that predict the THVLTS of the ACTIVE study.

## 4. Discussion

Based on the selected sample participants in the ACTIVE study dataset (Willis et al., 2015), the Bayesian hierarchical linear models of three types were fitted by considering only six explanatory variables as predictors of the cognitive verbal learning test. The null model without any predictor effect but with only the intercept term was fitted, and it shows a mass of cognitive verbal learning ability variability across subjects. The varying intercept model with the addition of all predictor variables was fitted; and getting booster training, age, and reasoning ability were significant predictor of verbal test scores (Duff, 2016). The varying coefficient/slope model (i.e., Model 3) is the best-fitted model than the other fitted models since it had the lowest WAIC, LOO-IC, and 10-fold estimates (Bafumi and Gelman, 2011). A bulk of participants’ cognitive verbal test scores variations were observed between subjects (Ryoo, 2011). The full hierarchical model with varying intercepts and slopes has the best performance for predicting verbal learning tests (from ACTIVE study dataset) using the hybrid Hamiltonian Markov Chain Monte Carlo approach.

Socio-demographic and training-related characteristics influence elderly verbal learning tests that can be measured in multiple occupations (Welsh-Bohmer et al., 2009).

## 5. Conclusion

Total Hopkins Verbal Learning Test Score from the ACTIVE study can be used as a measure of elderly adults’ cognitive verbal learning ability. Four demographic characteristics of adults, such as age, gender, educational status, and cognitive status (MMSE score), were measured at the baseline, and characteristics measured after cognitive training such as reasoning ability and booster training were considered. THVLTS from the ACTIVE study can be used as a measure of elderly adults’ cognitive verbal learning ability. According to the findings, the varying intercept and slope model fit best, and age, gender, booster, and reasoning ability are the main significant predictors for THVLTS, which measures cognitive verbal learning. Taking booster training had a positive significant predictive effect, while years of education (edu) had a negative significant predictive effect on THVLTS.

## Data Availability Statement

The datasets presented in this study can be found in online repositories. The names of the repository/repositories and accession number(s) can be found in the article/supplementary material.

## Ethics Statement

Ethical review and approval was not required for the study on human participants in accordance with the local legislation and institutional requirements. Written informed consent for participation was not required for this study in accordance with the national legislation and the institutional requirements. Written informed consent was not obtained from the individual(s) for the publication of any potentially identifiable images or data included in this article. This is because this quantitative analysis and modeling paper used open-access secondary data on repeated measurements.

## Author Contributions

EE participated in all aspects of the study: designing the study, performing data management, conducting the data analysis, writing the first draft of the manuscript, and discussing with MC to improve the manuscript, as it is a part of the first author’s Ph.D. dissertation. MC participated in revising the manuscript, commenting, and proofreading. Both authors listed have made a substantial, direct, and intellectual contribution to the manuscript and approved it for publication.

## Conflict of Interest

The authors declare that the research was conducted in the absence of any commercial or financial relationships that could be construed as a potential conflict of interest.

## Publisher’s Note

All claims expressed in this article are solely those of the authors and do not necessarily represent those of their affiliated organizations, or those of the publisher, the editors and the reviewers. Any product that may be evaluated in this article, or claim that may be made by its manufacturer, is not guaranteed or endorsed by the publisher.

## Appendix

### The Priors in Bayesian Hierarchical Models and Sensitivity Analysis

Different scholars suggested various priors for a component in a hierarchical model of variability parameters depending on the fitted Bayesian model structure and MCMC method. Some researchers proposed non-informative prior distributions, including uniform and inverse-gamma families in Gibbs sampling. Other researchers suggested a half-t family for the hierarchical model and demonstrate relatively weakly informative prior distribution. Half-student-t prior, a default prior in BRMS for SD parameters leads to better convergence. Still, the local shrinkage parameters lead to an increased number of divergent transitions in the BRMS of Stan (Piironen and Vehtari, 2016). The robust options for group-level standard deviations in Bayesian hierarchical models are half-normal (0,100), half-Student t with 3 degrees of freedom, or half-Cauchy prior distributions (Congdon, 2020).

Bürkner (2019) and McElreath (2020) proposed a half-normal distribution for SD priors in BRMS. Choosing a truncated normal distribution considers a good idea that the standard deviation cannot be less than 0. However, a prior on the random effect parameter with a long right tail has been revealed as “conservative” because it allows for bulky value estimates of the SD parameters.

Gelman (2006) suggested half-Cauchy prior with a mode at 0 and scale set to a considerable value by reasonably explaining the restrictive nature of half-Cauchy prior in providing enough information for the small numbers of groupings in the hierarchical nature of the data. Bayesian hierarchical model, to reduce the occurrence of unrealistically large SD estimates, BRMS-Stan documentation suggested half-Cauchy is the prior that automatically bound at 0. R-Stan renormalizes the distribution used so that the sum of the area between the bounds is 1.

The half-Cauchy (0, 1) prior is a case of the half-Student *t*-distribution with *v*1 degrees of freedom parameterized in the *SD* metric. It occupies a reasonable middle ground of different prior classes that performs well near the origin. It does not lead to drastic compromises estimates of the population-level (location) and group-level effect of the parameter space (Polson and Scott, 2012).

Sensitivity analysis of priors Appendix Table 1 shows the robustness of a Bayesian analysis when choosing different prior distributions in the fitted models. According to Bürkner (2019), for each SD component of the random parameters in hierarchical models, any prior distribution is practically well-defined on the non-negative real numbers only. In this study, we used the default in BRMS, the truncated Student’s t distribution with 3 degrees of freedom considered a reference prior. Then, because negative values are incredible for a standard deviation, we practiced a very strong informative truncated normal prior with a mean of 5 and a standard deviation of 0.01, and a half Cauchy prior for the sensitivity analysis of the impact of the prior on the Bayesian hierarchical models for the applied dataset.

Models with an effective sample size greater than 1000 and R-hat closest to 1.00 but not greater than 1.10 showed the consistency of an ensemble of Markov chains (Dominique, 2015). Moreover, both Bulk-ESS and Tail-ESS should be at least 100 (approximately) per Markov chain to be reliable and indicate that estimates of the respective posterior quantiles are reliable (Vehtari et al., 2021). In this paper, the R-hat, Bulk-ESS, and Tail-ESS results of the null, varying intercept, and varying coefficient models fulfilled these convergence diagnosis metrics. Therefore, the effective sample sizes (ESS) and potential scale reduction (R-hat) convergence diagnostic metrics are sufficient for stable estimates in each fitted model.

We used the sensitivity analysis for priors to scrutinize the final fully hierarchical specified model (Model 3) results, based on the default (or reference) prior, with the results obtained using different prior distributions. The posterior distributions and 95% posterior density (HPD) intervals by the median for the fixed effects and random effects, including SD of the verbal learning test score, did not change much depending on the priors specified, which indicates a practically identical interpretation of the estimates depending on the priors. Thus, because of no significant percentage deviation among models depending on the alternative prior specification, we reported the model results with the half-Cauchy prior that yielded good model convergence and sufficient ESS values (i.e., greater than or equal to 100). Besides, a good-looking posterior density plot of the predicted versus the observed data in Figure 4 was considered.

As it can be explained by Depaoli and van de Schoot (2017), and Depaoli et al. (2020), sensitivity analysis results could be provided through visuals, akin to the Shiny app plots or it may be in a table format indicating the degree of discrepancy in estimates or HPD intervals across parameters as we presented in the Appendix table below.

**APPENDIX TABLE 1:** Posterior estimates with the verity of priors: Sensitivity analysis results.

Alternative priors	Parameter/Covariates	Estimate (SD)	Median (50%)	5–95% HDP	Default estimate (SD)	Percentage deviation
Alternative prior I: Half- Cauchy (0,1)	Intercept	9.8412 (1.521)	9.8331	5.8523, 13.9344	9.8321 (1.932)	0.0926

	Age	−0.1211 (0.021)	−0.1201	−0.1513, −0.0823	−0.1223 (0.423)	−0.9812
	Edu (education)	−0.0033 (0.001)	−0.0033	−0.0122, 0.0012	−0.0034 (0.005)	−2.9412
	Booster	0.1412 (0.102)	0.1413	−0.2145, 0.4831	0.1411 (0.623)	0.0709
	Gender	2.5505 (0.112)	2.5504	2.1712, 2.9331	2.5487 (0.222)	0.0706
	Reason	0.1444 (1.902)	0.1443	0.1313, 0.4402	0.1443 (2.081)	0.0693
	MMSE	0.5803 (0.028)	0.5921	0.4822, 0.6840	0.5801 (0.082)	0.0345
	σ_*intercept*,*subject*_	1.9222	1.9221	0.0724, 4.3111	1.9212	0.0521
	σ_*intercept*,*time*_	1.3022	1.3102	0.5702, 3.1343	1.3032	−0.0767
	σ_*age*_	0.0424	0.0403	0.0123, 0.0625	0.0425	0.0126
	σ_*reason*_	0.0405	0.0402	0.0212, 0.0732	0.0401	−0.9975
	*cor*_*Intercept*,*age*_	0.1033	0.1032	−0.7042, 0.8303	0.1031	0.1040
	*cor*_*Intercept*,*reason*_	−0.3902	−0.8902	−0.9011, 0.6212	−0.3904	−0.0512
	*cor*_*age*,*reason*_	−0.5922	−0.5887	−0.9042, 0.1407	−0.5923	−0.0169
	σ_*e*_(*sigma*)	3.1102	3.2041	3.0533, 3.1710	3.1112	−0.0321
	Parameter	Estimate (SD)	Median (50%)	5–95% HDP	Default estimate (SD)	Percentage deviation

Alternative prior II: Normal (5, 0.01)	Intercept	9.8423 (1.543)	9.8231	6.1415, 13.6552	9.8321 (1.932)	−0.0112

	Age	−0.1212 (0.034)	−0.1212	−0.1514, −0.0855	−0.1223 (0.423)	−0.0825
	Edu (education)	−0.0034 (0.011)	−0.0034	−0.0124, 0.0015	−0.0034 (0.005)	−2.9412
	Booster	0.1413 (0.124)	0.1412	−0.2165, 0.4871	0.1411 (0.623)	−0.0708
	Gender	2.5514 (0.142)	2.5505	2.1722, 2.9371	2.5487 (0.222)	−0.0353
	Reason	0.1445 (2.013)	0.1444	0.1453, 0.4562	0.1443 (2.081)	−0.0692
	MMSE	0.5802 (0.035)	0.5872	0.4852, 0.6951	0.5801 (0.082)	0.0172
	σ_*intercept*,*subject*_	1.9213	1.9221	0.0724, 4.3413	1.9212	0.0468
	σ_*intercept*,*time*_	1.3033	1.3102	0.5622, 3.1344	1.3032	−0.0844
	σ_*age*_	0.0425	0.0403	0.0123, 0.0627	0.0425	−0.2353
	σ_*reason*_	0.0406	0.0402	0.0212, 0.0733	0.0401	−0.2463
	*cor*_*Intercept*,*age*_	0.1034	0.1032	−0.7044, 0.8304	0.1031	0.0969
	*cor*_*Intercept*,*reason*_	−0.3903	−0.8902	−0.9021, 0.6217	−0.3904	−0.0256
	*cor*_*age*,*reason*_	−0.5923	−0.5987	−0.8045, 0.1404	−0.5923	−0.0169
	σ_*e*_(*sigma*)	3.2115	3.2141	3.0533, 3.5710	3.1112	−3.1543

*The relative percentage deviation can be computed as: {[(estimate using new alternative prior)–(estimate using default/reference prior)]/ (estimate using default/reference prior)}*100. Interpreting percentage deviation results is largely subjective and dependent on the metric of the parameters. However, percentage deviation under 10% would likely be considered negligible (Depaoli and van de Schoot, 2017). Default estimate = posterior estimate (mean) of analysis with the BRMS default/reference prior [Student’s t (3); 5–95% is the highest posterior density (HPD) interval].*
